# Disentangling Responsibility: Perspectives on Dementia Prevention From Stakeholders in Canada, Germany and Switzerland

**DOI:** 10.1111/1467-9566.70126

**Published:** 2025-12-08

**Authors:** Niklas Petersen, Mattia Andreoletti, Alessandro Blasimme, Cynthia Lazzaroni, Annette Leibing, Silke Schicktanz

**Affiliations:** ^1^ Department of Medical Ethics and History of Medicine University Medical Center Göttingen Göttingen Germany; ^2^ Department of Health Sciences and Technology ETH Zürich Zürich Switzerland; ^3^ Department of Social Studies of Medicine McGill University Montreal Quebec Canada; ^4^ Faculty of Nursing University of Montreal Montreal Quebec Canada

**Keywords:** dementia, health policy, health promotion, prevention, responsibility

## Abstract

Dementia prevention has become a priority in medical research and public health. Various medical, social and environmental risk factors have been identified; however, medical studies, media discourses and health policies predominantly centre on individual‐level prevention and promote personal responsibility for maintaining cognitive health. This emphasis on individual preventive efforts faces increasing criticism for being ethically problematic, medically insufficient and reflective of a neoliberal responsibilisation of (cognitive) ageing. Our study, based on 60 semi‐structured qualitative interviews with stakeholders involved in dementia research, care and health policy from Canada, Germany and Switzerland, situates the preventive turn within contemporary ageing cultures and welfare policies. By examining the epistemic and normative arguments underlying current debates on dementia prevention, we illustrate how stakeholders justify both personal and governmental responsibilities in preventing dementia. Although managing and mitigating various risk factors throughout life is often framed as a matter of personal responsibility, many stakeholders advocate combining individual‐level prevention with population‐level efforts to address social inequalities that affect the risk of developing dementia. Drawing on the interview study, we highlight the importance of recognising the political and normative foundations and implications of dementia prevention strategies.

## 1 Introduction

Dementia, once seen as an unpreventable condition, is now widely recognised as a partly avoidable syndrome and has become a focal point of medical and public health preventive efforts. Epidemiological research continuously accumulates evidence about the impact of various risk factors and advocates for measures to reduce the risk of dementia (Livingston et al. 2024). This new preventive paradigm has been adopted by many national dementia strategies, which recognise, to varying degrees, dementia prevention and early interventions as fundamental goals (Andreoletti and Blasimme 2025; Godard‐Sebillotte et al. 2024; Hampel et al. 2022).

Although recent epidemiological studies have identified a large variety of medical, lifestyle‐associated, social and environmental risk factors for dementia throughout the life course, policymaking, research and media discourses tend to focus on individual‐level interventions (Daly 2024a; Walsh et al. 2024; Petersen and Schicktanz 2021). The dominant emphasis on individual preventive efforts and personal responsibility in medicine, public health and media discourses is increasingly criticised for being therapeutically inadequate and ethically problematic (Daly 2024b; Forlini and Hall 2017; Walsh et al. 2022; Wilson and Anstey 2024).

This study, therefore, aims to extract and differentiate the underlying foundations and justifications implied in current dementia discourses. In the background section, we outline the preventive turn in dementia research (Section 2.1), discuss epidemiological, sociological and ethical debates on dementia prevention (Section 2.2) and describe the different ways in which prevention is addressed in Canadian, German and Swiss health policies and dementia strategies (Section 2.3). Subsequently, we outline our methodological approach, drawing on an interview study with stakeholders engaged in dementia research and healthcare in Canada, Germany and Switzerland (Section 3). In the results section, we examine how stakeholders assess the relevance of various risk factors for dementia and whom they address based on which epistemic, pragmatic and normative arguments as being responsible for preventing dementia (Section 4). Finally, we discuss stakeholder perspectives in the context of current health policies. We argue for the need to focus on the social, economic and environmental dimensions of risk factors and to acknowledge the political and normative underpinnings and implications of prevention approaches (Section 5).

## 2 Background

### 2.1 The Turn Towards Prevention

Given the lack of effective curative therapy^1^ for dementia and the social, ethical and economic challenges associated with rising dementia rates, attention in medical research and health policy debates has shifted towards the potential of dementia prevention (Andreoletti and Blasimme 2025; Forlini and Hall 2017; Hampel et al. 2022). Building on the estimation that 45% of dementia cases could be avoided by targeting 14 modifiable risk factors (Livingston et al. 2024), dementia prevention is conceptualised as a lifelong project: The preventive endeavour begins with good education in youth. Risk reduction in middle age includes avoiding smoking and excessive alcohol, reducing high cholesterol by adopting a healthy diet and staying physically active, as well as treating conditions such as hearing loss, hypertension, obesity and depression. Relevant risk factors in later life are social isolation and exposure to air pollution (Livingston et al. 2024).

Most existing medical intervention studies and health policy approaches towards dementia prevention, however, tend to favour individual‐level measures and highlight the importance of sufficient cognitive, physical and social activity and a healthy diet for maintaining cognitive functioning until old age (Daly 2024a; Forlini and Hall 2017; Walsh et al. 2024). Even if national dementia strategies mention social and health inequalities or environmental factors, they often lack tangible measures and objectives to reduce them (Godard‐Sebillotte et al. 2024). In the media and public discourses in many Western countries, preserving cognitive abilities into old age is often portrayed primarily as the outcome of a healthy and active lifestyle, framed as an individual's personal responsibility (Chelberg 2025; Lawless and Augoustinos 2017; Petersen and Schicktanz 2021; Šestáková and Plichtová 2020). Despite optimism based on the Lancet reports that a significant proportion of dementia cases could potentially be prevented, it must be noted that the actual scale of the preventive potential remains controversial (Kivimäki and Singh‐Manoux 2018), the causal link between many risk factors and dementia remains uncertain (Desai et al. 2023), and many risk factors are not easily modifiable and behavioural changes might be improbable in practice (Leanza 2020).

### 2.2 Epidemiological, Sociological and Bioethical Debates on Dementia Prevention

The focus on individual‐level prevention and personal responsibility in current dementia discourses has been controversially discussed from epidemiological, sociological and normative perspectives. As we will briefly outline below, the debate comprises three main stances. First, epidemiologists problematise the current focus on individual‐level prevention in research and health policy for neglecting social, economic and environmental determinants of health risks and thus being ineffective. They highlight the importance of population‐level risk factors and emphasise the need to focus more strongly on structural interventions to reduce the prevalence of dementia (Daly 2024b; Mukadam et al. 2024; Walsh et al. 2022). Measures discussed include policy changes that aim to induce unconscious or involuntary behaviour changes (through, e.g., smoking bans and taxes), initiatives to improve detection and management of known medical risk factors such as hypertension and diabetes but also structural interventions aimed at increasing living and working conditions and reducing environmental risk factors by, for example, reducing air pollution (Mukadam et al. 2024; Walsh et al. 2022).

Second, scholars in medical sociology and critical gerontology have analysed the prevention paradigm—particularly its emphasis on individual‐level prevention—in its embedding in neoliberal governance (Ayo 2012; Brown and Baker 2013) and anti‐ageing ideology (Cardona 2008; Schweda and Pfaller 2014), arguing that the responsibility to maintain cognitive health until old age is shifted to the individual (Chelberg 2025; Foth 2020; Latimer 2018; Schweda and Pfaller 2020). The call for individual behavioural prevention aligns with the logic of activating social policy, which places responsibility on the individual to avoid becoming a burden on the welfare state (van Dyk and Lessenich 2009). Cognitive decline would then be ideologically framed as a personal failure, whereas the preservation of cognitive performance into old age would function as a cultural signifier of responsible citizenship and successful ageing (Petersen and Schicktanz 2021; Katz 2012; Williams et al. 2012). The discursive framing of dementia as a result of ‘irresponsible’ lifestyle choices could lead, as some have argued, to victim‐blaming and additional stigmatisation of those affected (Horstkötter et al. 2021; Peel 2014; Wilson and Anstey 2024). As a result of public health messaging that emphasises the importance of lifestyle risk factors, laypeople feel, as Peckham et al. (2024) have shown based on a qualitative interview study, a personal responsibility to control their dementia risk, which may contribute to the stigmatisation of dementia as a lifestyle disease.^2^

However, in response to criticisms regarding the emphasis on personal responsibility for health and cognitive ageing, some bioethicists and sociologists argue that a blanket rejection of attributing personal responsibility for prevention and lifestyle choices falls short. Taking responsibility for one’s health and exercising moral agency could, as, for example, medical sociologist Rier (2022) argues, increase dignity and improve health and autonomy even in unequal social structures. Given the rising prevalence of lifestyle‐related diseases, some liberal and utilitarian ethicists propose holding individuals accountable for their choices and even suggest using personal responsibility as a criterion for allocating limited resources (Buyx 2008).

This article argues that rethinking current approaches to dementia prevention requires a precise understanding of responsibility and a thorough assessment of the medical, pragmatic and normative arguments that underscore the importance of different approaches to dementia prevention and the respective roles of personal and governmental responsibilities. We follow Schicktanz and Schweda’s (2012) relational conceptualisation of responsibility as a multirelational meta‐ethical concept, enabling a systematic analysis of responsibility ascriptions that considers the relationship between the moral agent and the object, the underlying norms, as well as the causality of action and its outcomes. Using this framework and drawing on our interview study, we examine how Canadian, German and Swiss stakeholders assess the evidence for and strengths of the causal relationship between lifestyle‐associated, social and economic factors and the risk of developing dementia, and we differentiate the argumentative patterns they use to justify personal and governmental responsibilities in dementia prevention.

### 2.3 Dementia Prevention in Canada, Germany and Switzerland

We chose these three Western industrialised countries for comparison, as they all have solidarity‐based healthcare systems but differ in terms of funding (tax‐funded vs. insurance‐based), the scope of health services covered and the balance between private and public sponsorship of health facilities. Moreover, the importance of health promotion in healthcare has historically differed considerably between Canada, Germany and Switzerland, and the current national dementia strategies show a notable discrepancy in the emphasis placed on prevention.

In Switzerland and Germany, prevention and health promotion have traditionally played a minor role in the healthcare system. However, in both countries, reforms over the past decade have aimed to strengthen prevention, primarily to limit healthcare costs and enhance the population's ability to work (Lückenbach et al. 2023). The German Prevention Act of 2015 aimed to improve cooperation among health insurance providers, states and municipalities to promote health and disease prevention. The German healthcare system is nonetheless still commonly criticised for its emphasis on curative care while spending very few resources on prevention and health promotion (Zeeb et al. 2025). In 2020, Germany adopted a National Dementia Strategy aimed at improving the care and daily life of people with dementia. The strategy focuses on promoting social participation, providing better support and counselling, improving medical care and nursing services, and advancing research (BMFSFJ and BMG 2020). Although health campaigns aim to increase awareness of lifestyle‐associated risk factors, critics note that there is still a lack of focus on early diagnosis, support during the early stages of the disease and systematic efforts to strengthen prevention (Jessen 2022). The Swiss National Dementia Strategy also prioritises care over prevention. Despite describing risk reduction as crucial, it lacks specific references to measures that translate medical evidence into policies and healthcare practices (Andreoletti and Blasimme 2024).

In contrast, in Canada, the significance of prevention in health policy has been recognised since the 1974 Lalonde Report, which acknowledges the impact of socioeconomic and environmental factors on health but mainly emphasises promoting individual lifestyle changes to reduce health risks (Martin et al. 2018; Foth 2020). In Canada’s national dementia strategy, released in 2019, prevention is one of three main objectives (Public Health Agency of Canada 2019). Besides aiming to improve the quality of life of people living with dementia and advance therapies for dementia, the strategy focuses on fostering research on modifiable risk factors, expanding awareness of risk factors and effective interventions, promoting the adoption of effective interventions and supporting measures that promote healthy lifestyles through adapted social and physical environments.

Assuming that these different public health traditions, policy frameworks and national dementia strategies influence how the preventive turn in dementia research translates into local understandings and prevention methods, the BEAD study examines how stakeholders in Canada, Germany and Switzerland perceive the changing understanding of dementia and how they negotiate challenges and social, ethical and medical implications of dementia prevention.

## 3 Methods

The analysis is based on an interview study with stakeholders from central fields of dementia prevention and care. Recognising that the understanding of dementia and views on prevention strategies can vary depending on occupational role, professional experience and involvement with dementia care and prevention, we included a diverse group of stakeholders in our study. This group comprised researchers in dementia‐related fields (neurosciences, neurology and geriatrics), healthcare providers (physicians and nurses), patient advocates (e.g., Alzheimer’s Association and local self‐help groups), technology developers, bioethics professors, as well as representatives from health ministries, local health authorities and insurance companies (see Table 1). With various scientific and institutional backgrounds, these stakeholders provide diverse insights into the medical, social, ethical and political aspects of preventive strategies, enabling us to capture a comprehensive view of the ongoing debate surrounding dementia prevention. Stakeholders were recruited based on their role in current dementia discourse, their publications and peer recommendations. We conducted interviews with 64 stakeholders via Zoom or phone in 2022 and 2023.

**TABLE 1:** Overview of the sample.

Professional background	Canada (Can)	Germany (Ger)	Switzerland (Sui)
Patient advocacy (Adv)	5	4	3
Politics (Pol)	4	3	1
Economy (Eco)		2	1
Ethics (Eth)	2	3	1
Technology development (Tech)	7	3	3
Science (Sci)	5	4	2
Healthcare (Health)	2	4	5

We developed a semi‐structured interview guide based on preliminary literature research and an analysis of medical, nursing and public discourse on dementia prevention (Petersen and Schicktanz 2021). The structured questions aimed to collect experts’ knowledge on dementia risk factors, international differences in dementia prevention and public health policies, responsibility ascriptions for dementia prevention and views on the role of current and future technologies (see Supporting Information S1). All participants received a detailed explanation of the study objectives and procedures, were assured anonymity and confidentiality, and signed an informed consent form. Our study was approved by the Ethics Committees of the ETH Zürich, the University of Montreal and the University Medical Center Göttingen. To protect the confidentiality and anonymity of the participants, all identifying information was removed from the transcripts, and pseudonyms were used when referring to participants. The interviews were conducted in English, German or French and lasted between 40 and 90 minutes, were transcribed verbatim and were translated to English. We used the QDA software ATLAS.ti® to analyse the anonymised interviews from each country, employing a qualitative content analysis approach (Kuckartz and Rädiker 2023). Main categories were developed deductively based on preliminary literature research and discourse analysis and were supplemented inductively during the analysis.

## 4 Results

In the following analysis, we highlight the normative, epistemic and pragmatic justifications that stakeholders present to emphasise the importance of individual‐level versus population‐level preventive measures. We describe the variety of normative, epistemic and pragmatic arguments that explain the differing positions regarding responsibilities for dementia prevention (see the overview in Table 2), using quotes from stakeholders from all countries, and do not offer a systematic comparison between countries or professions. Because of limited space, we present only selected stakeholder excerpts, acknowledging that similar reasoning was often expressed across countries and professions.

**TABLE 2:** Epistemic, normative and pragmatic arguments for ascribing responsibility for dementia prevention.

Position on responsibility ascriptions	Epistemic foundations	Normative justifications	Pragmatic and economic reasoning
Defending personal responsibility for individual‐level prevention (see Section 4.1)	Current epidemiological evidence indicates that physical, cognitive and social activities reduce the risk of developing dementia.	Healthy lifestyle choices and proactive preventive efforts are a civic duty.	Individual‐level prevention is the only option, given the failure of governmental health policies.
		Lifestyle choices are a matter of individual freedom.	
Questioning the focus on personal responsibility and individual‐level prevention (see Section 4.2)	The focus on individual‐level prevention neglects relevant socioeconomic and environmental risk factors.	Individualising responsibility ascriptions poses the risk of stigmatisation and contradicts egalitarian values.	Lifestyle changes for preventing dementia are improbable in practice.
Emphasising governmental responsibility for population‐level prevention (see Section 4.3)	Given the current epidemiological evidence, population‐based interventions are essential for tackling the most significant risk factors.	Addressing socioeconomic risk factors is essential for achieving health equity.	Increased governmental efforts are economically necessary due to the increasing disease burden.

Drawing from the interviews, we first identify three distinct reasoning patterns interviewees used to underscore the significance of individual preventive efforts and personal accountability (Section 4.1). Next, we examine how other stakeholders question this individual‐centric approach (Section 4.2), and we present the arguments they use to emphasise the necessity of population‐level interventions and the relevance of governmental responsibility in dementia prevention (Section 4.3). Lastly, we explore two opposing approaches regarding how the interviewed stakeholders linked personal and governmental responsibilities in the context of dementia prevention (Section 4.4).

### 4.1 Justifications for Personal Responsibility for Individual‐Level Dementia Prevention

The focus on individual‐level prevention and personal responsibility prevalent in public and health policy discourses (Chelberg 2025; Lawless and Augoustinos 2017; Petersen and Schicktanz 2021) was reflected in some interviews. Below, we differentiate between evidence‐based foundations (Section 4.1.1), neoliberal and libertarian justifications (Section 4.1.2) and pragmatic reasoning patterns (Section 4.1.3) that stakeholders use to defend the framing of dementia prevention as a personal choice.

#### 4.1.1 Epistemic Foundations for Individual‐Level Dementia Prevention

The interviewed stakeholders acknowledged a significant increase in the understanding of the aetiology and biology of dementia and referred to a paradigmatic shift towards prevention in dementia research. The stakeholders frequently referred to the *Lancet Commission* reports on dementia prevention (Livingston et al. 2024), agreeing that over the past decade, the scientific evidence underpinning the potential for dementia prevention has expanded considerably. ‘Things have changed, in my opinion, because we have accumulated enough evidence—solid data—that has allowed us to draft robust guidelines. At this point, I mainly refer to the paper from the Lancet Commission’ (SuiAdv1^3^).

Based on the increasing evidence for the potential of dementia prevention, several stakeholders emphasised the importance of an active lifestyle to reduce the risk of developing dementia. ‘That is social participation, that is exercise, where we know that it is incredibly important in old age and to be curious about life, to learn new things, to discover new content’ (GerAdv3). Mirroring gerontological and political conceptions of active and successful ageing (Rowe and Kahn 1997; Walker 2002), staying physically and cognitively active, as well as socially engaged, is considered crucial to maintaining health and cognitive functioning in old age. Based on the understanding of the preventive effects of an active lifestyle, some stakeholders frame staying active, as the following interview excerpt indicates, as an imperative throughout the life course:

Regular physical exercise that's super, super important; keeping your brain active, that's super important. But if it's good for your heart, it's good for your brain. And regular physical activity, it's good for your heart. […] I think controlling health risks early and being proactive about health is kind of the rule, no matter what the age is.

(CanHealth2)

For many stakeholders, activity is a central value related to preventing dementia and, more broadly, ageing well. However, as we will show below, the normative justifications for framing dementia prevention as a matter of personal responsibility and a proactive lifestyle varied considerably.

#### 4.1.2 Normative Justification for Personal Responsibility for Dementia Prevention

A German health official argued that the ‘responsibility for a healthy lifestyle lies with every individual’ (GerPol2). Rejecting governmental responsibility for dementia prevention, he called on individuals to adopt a health‐conscious, preventive lifestyle:

If I know how to live healthily, then in principle, from my point of view, I am obliged as a citizen to behave accordingly. That's why not the state, not the health insurance companies, has the responsibility—it's the individual citizen's responsibility to begin with. And I don't need any support measures in principle.

(GerPol2)

In the context of this authoritative call to adopt a proactive lifestyle, prevention is framed not only as a social obligation but is explicitly supported by the claim that a healthy lifestyle does not require governmental or social backing. Similarly, a Canadian health practitioner argued that people must take responsibility for their health to prevent dementia: ‘I feel like we have to be responsible for our own health, first and foremost. I think we can’t rely, in any situation, on others to be responsible for us. I think people do need to take personal responsibility and be proactive about their health’ (CanHealth2). The notion of an ‘obligation’ suggests that the call to adopt an active lifestyle to prevent dementia is understood not only in terms of individual freedom and as a chance to preserve one’s autonomy and quality of life into old age but also as linked to the idea of a social duty to avoid becoming a burden on the health system and society. In line with neoliberal social and health policies that privatise risks and hold individuals responsible for maintaining their (cognitive) health until old age (Latimer 2018; Chelberg 2025), stakeholders from all three countries and with diverse professional backgrounds address citizens as responsible for adopting healthy lifestyles in order to prevent dementia.

However, in contrast to the notion of dementia prevention as an individual duty, other stakeholders from Canada and Switzerland employed alternative patterns of reasoning to frame dementia prevention as a matter of personal lifestyle choices. For example, some interviewees framed decisions for or against a health‐conscious, preventive lifestyle not as a moral question of good citizenship but as a matter of individual freedom. A Swiss bioethicist argued: ‘I believe that the responsibility is on the individual and that the state should intervene only when individual freedom harms others. I think each person has the right to manage their own cognitive health as they wish’ (SuiEth1). In this context, individuals are understood to be free to make their own choices regarding cognitive health without state intervention, unless their actions harm others. Bad lifestyle choices that increase the risk of dementia should, as also suggested by a Canadian bioethicist, be recognised as part of individual autonomy: ‘I mean, people can make bullish choices if they want to; that's part of their liberty, right? People can smoke, they can drink a lot of alcohol. If they want to do something that increases the risk of dementia, I suppose that’s their right’ (CanEth2).

Within this libertarian framework (Flanigan 2022), governmental interventions are viewed as paternalistic, and personal health decisions are regarded as a matter of individual freedom; governmental responsibility is restricted to protecting individual rights. In contrast to the neoliberal notion of a citizen’s duty to prevent dementia, individual choices to engage—or not engage—in dementia prevention are perceived as personal freedom rather than a moral obligation.

#### 4.1.3 Pragmatic Reasoning for Individual‐Level Prevention

In addition to these two normative justifications for emphasising individual preventive efforts, some stakeholders highlighted the pragmatic argument that due to the shortcomings of public health prevention efforts, enhancing individual‐level prevention remains the last viable option for reducing dementia rates.

You can give money to the government, but honestly, they may not be doing their job. And you're talking about prevention, not treatment. You must go back to the individual. That's why I invest so much of my time in public conferences and the media. It's a lot of energy, but I think that's where it's having an impact today.

(CanSci4)

It appears that for some stakeholders, the failures of public institutions to adequately provide for dementia prevention—such as healthy living conditions, access to good healthcare and education for healthcare providers and the public—result in individuals facing a greater responsibility for their own healthcare. In light of the difficulties in translating knowledge on dementia risk factors into policy (Godard‐Sebillotte et al. 2024; Andreoletti and Blasimme 2024), they identified strengthening awareness of the potential of individual‐level efforts and lifestyle changes as the only viable option for advancing dementia prevention.

### 4.2 Objections to the Focus on Individual‐Level Prevention and Personal Responsibility

The understanding of dementia prevention as a matter of individual effort and lifestyle choices was, however, not unanimously shared among stakeholders. Interviewees from all countries and professional backgrounds criticised the emphasis on individual‐level prevention as medically ineffective (Section 4.2.1), potentially increasing stigmatisation (Section 4.2.2) and improbable in practice (Section 4.2.3).

#### 4.2.1 Epistemic Objections to Individual‐Level Prevention

Although a broad agreement and increasing confidence in the potential of dementia prevention were evident in our sample, the stakeholders' assessments regarding the strengths of evidence and the effectiveness of specific preventive measures differed considerably. Some stakeholders problematised unclear or weak medical evidence for the effectiveness of specific measures and stressed that the epidemiological models would have little predictive value at the individual level. Not doubting the possibility of dementia prevention per se, some stakeholders, for example, a German neuroscientist, problematised a premature conclusion from correlation to causality and pointed to contradictory studies on specific risk factors and preventive measures:

An observed correlation is not yet causality. And the correlation between the duration of education and the frequency of later dementia is a correlation. Turning it into a causality does not necessarily lead to the desired result of less dementia. […] Physical activity, for example, there is a lot of epidemiological evidence, but to date, no clinical study can show that there is really a lasting long‐term improvement or even dementia prevention.

(GerSci1)

As this interview exemplifies, several stakeholders from the scientific community have urged caution against specific recommendations and generalisations on the effects of preventive interventions. Noting that models developed from epidemiological studies do not allow for conclusive claims on actual causality between individual risk factors and dementia (Desai et al. 2023), they highlighted the lack of (and challenges to) clinical efficacy studies demonstrating the effectiveness of specific preventive interventions. The interviewees referred to various, sometimes contradictory, studies (see, e.g., Brasure et al. 2018; Kivimäki and Singh‐Manoux 2018), and it appeared that, to date, no consensus exists on the strength and quality of the evidence for dementia prevention.

#### 4.2.2 Normative Objections to Individualising Responsibility

In addition to these epistemic objections to an excessive focus on individual responsibility for dementia prevention, some stakeholders argued that moralising responsibility ascriptions would be ethically problematic, as they contradict egalitarian values and might support the stigmatisation of people living with dementia. Despite recognising the relevance of individual prevention efforts and lifestyle choices, a German neurologist rejected the idea of a duty to adopt a preventive lifestyle as being contradictory to societal values. ‘With lifestyle, it depends on the individual. You can say that this is also a responsibility in the sense of an obligation. […] [However,] I don’t think you can somehow turn it into a sanctionable responsibility in the sense of a duty. Yes, our society is not structured like that at all’ (GerHealth1). Similarly, an ethicist argued against ascribing responsibility or even fault linked to lifestyle choices, as tolerating even unhealthy lifestyles was part of our pluralistic society.

I think we have to avoid ascriptions of backwards‐looking responsibility. When people have a particular type of health outcome and say, well, you are responsible for the dementia you have developed because all your life you have been drinking and eating and doing the wrong things in general in terms of physical activity, that's really your own fault. I think that's something that we have to avoid. Because in the end, we are a society that accepts and tolerates a plurality of lifestyles, even unhealthy ones.

(GerEth1)

Furthermore, engaging with debates concerning the stigmatisation of preventable diseases (e.g., Best and Arseniev‐Koehler 2023; Wilson and Anstey 2024), stakeholders from all countries raised concerns that framing dementia as an avoidable lifestyle disease might change attitudes towards those affected by dementia.

Because if we see the media telling the population that dementia is avoidable, this could possibly change our attitude against dementia patients. Because if you have somebody in front of you who is sick from a disease that you think is avoidable, then you think, maybe subconsciously, that it’s his own fault.

(GerSci4)

Hinting at the psychological burden of understanding dementia as self‐inflicted and the possibility of sanctions and stigmatisation as a result, they urged caution against overemphasising personal responsibility. ‘It's an ethical problem that individual responsibility can be overemphasised and that, on that basis, people might be sanctioned if they have bad health outcomes in terms of neurodegenerative decline. (And this) may lead people to experience a particular psychological burden’ (GerEth1). As indicated in these interviews, some stakeholders noted a tendency towards simplification and exaggeration in media discourse on dementia (Petersen and Schicktanz 2021) and advocated for dementia risk communication that avoids simplified attributions of responsibility for dementia.

#### 4.2.3 Pragmatic Objections to Individual‐Level Prevention

Furthermore, several stakeholders were sceptical regarding the practical prospects of individual‐level prevention, questioning whether people would indeed adopt a healthy lifestyle based on the insight of the correlation between certain habits and the risk of developing dementia. ‘If the population gets to know that, in part, [dementia] is avoidable and that everybody can do something, I am being quite realistic and pessimistic that this doesn’t really change the health habits of the population’ (GerSci4). In particular, behavioural changes in later life aimed at delaying cognitive decline after the onset of dementia symptoms are viewed as challenging to implement, as one Canadian health practitioner describes: ‘Sure, this has a potential, although most of the patients are simply not able to change dramatically their life habits’ (CanHealth2). Although individual‐level prevention may be theoretically effective, several stakeholders consider its practical and effective implementation highly unlikely because medical evidence on dementia risks and information on preventive lifestyles do not necessarily lead to individual behavioural changes.

### 4.3 Justifications for Population‐Level Prevention and Governmental Responsibility

Many stakeholders from all three countries argued for the need to strengthen population‐level perspectives in dementia prevention. In our analysis, we distinguished between evidence‐based (Section 4.3.1), normative equity‐oriented (Section 4.3.2) and economic justifications (Section 4.3.3) for the importance of structural interventions in dementia prevention.

#### 4.3.1 Epistemic Foundations for Population‐Level Prevention

Stakeholders who assigned responsibility for dementia prevention mainly to the government highlighted that individual behavioural changes alone are insufficient to modify dementia risk factors effectively. They emphasised that community efforts are more effective than individual actions. A Swiss scientist pleaded for population‐based interventions, which, in view of the current evidence, would be more effective in reducing dementia rates:

The Post‐Thatcherian approach of empowering the individual to determine their own health, both diagnostically, therapeutically, and preventively, with dementia, is completely bankrupt. […] It is simply that our numbers show that the margin by which the individual can increase preventing his or her own dementia is almost negligible compared to what is achieved by community interventions.

(SuiSci1)

By describing the current focus on individual health decisions as ‘Post‐Thatcherian’, the stakeholder criticised the focus on individual‐level preventive efforts shaped by neoliberal ideology and argued further that according to current epidemiological evidence, the margin by which individuals can prevent dementia is almost negligible compared to what is achieved by community interventions. Discussing the current evidence on dementia risk factors (e.g., Livingston et al. 2024), several stakeholders argued that population‐based approaches towards dementia prevention would be the most promising way to reduce dementia rates.

#### 4.3.2 Normative Justifications for Governmental Responsibility for Prevention

Furthermore, the need to strengthen governmental efforts for population‐level approaches towards dementia prevention was justified normatively, as this would be crucial to achieving social and health equity. In this context, stakeholders discussed dementia prevention as a matter of equal health chances, access and equity. A Canadian bioethicist argued that health policy should prioritise making healthy living accessible instead of merely encouraging individuals to adopt lifestyle changes that are not easily accessible and financially feasible for everyone:

If I'm the minister of health, I think I need to be very careful about where I put the responsibility, that my messaging to Canadians and citizens is not, ‘It's your job to buy expensive salmon once a week and get a gym membership and take time off of work to go for a run’. I think we need to look at community‐level prevention and how we can facilitate these behaviours on a community level […]. So, my priority is to make the wonderful lifestyle changes that we know are effective and more accessible.

(CanEth1)

Especially in Canada, many stakeholders emphasised not only the relevance of socioeconomic factors but also of racial discrimination. Referring to research showing the increased risk of developing dementia in Indigenous and immigrant communities, a Canadian patient advocate emphasised the importance of considering diverse backgrounds and living conditions to strengthen dementia prevention in the various communities: ‘Research shows that racialised communities are disproportionately affected or at higher risk of getting dementia than non‐racialised communities, and Indigenous communities as well’ (CanAdv2). Several more interviewees stressed the importance of more targeted research, policy interventions and education campaigns tailored towards the needs of minorities. ‘For example, Indigenous populations in Canada have a higher risk of developing dementia. […] In Canada, inequality and diversity inclusion should be addressed […] I would say, again, more awareness, more research’ (CanTech7). In line with recent scientific debates on dementia prevention (Walsh et al. 2022; Wilson and Anstey 2024), these stakeholders did not view preventive efforts as an isolated project but discussed dementia prevention as part of the broader issues of health equity and emphasised the importance of reducing social and health disparities.

#### 4.3.3 Economic Reasoning for Population‐Level Prevention

In addition to these normative and evidence‐based arguments, stakeholders warned that without increased support from the state, the rising number of dementia cases would overwhelm the healthcare system. Taking up the economic reasoning that frames the introduction of preventive policies in Germany and Switzerland (Lückenbach et al. 2023), a Swiss patient advocate implied that increased health policy efforts to reduce dementia rates are a demographic and economic necessity: ‘Well, I personally think that the state has a very big responsibility. […] So, we are not doing enough. And it will be a big problem because the population is increasing and people are getting older. And we will have many more people with dementia’ (SuiAdv2). A Canadian technology developer also framed population‐level preventive approaches as a way to reduce healthcare costs in the (economic) interests of the whole society: ‘It's a good fiscal case as well, keeping the population healthier for longer. Ultimately, from an economic perspective, it also reduces health costs’ (CanTech5).

Above all, based on the insight that the focus on individual prevention and responsibility is insufficient due to epidemiological evidence, normative equity‐oriented and economic reasons, stakeholders from Canada, Germany and Switzerland, from diverse professional backgrounds, emphasised the need for increased government efforts in dementia prevention.

### 4.4 Joint Responsibility for Dementia Prevention

Most interviewees perceived dementia prevention as a shared endeavour. Nevertheless, the weighting of individual and governmental responsibility varied considerably. Shaped by the different normative foundations we have already identified, along with conceptions of health policy and assessments of medical evidence, two contrasting understandings of joint responsibility were prevalent among the interviewees: the informing state (Section 4.4.1) and the enabling state (Section 4.4.2).

#### 4.4.1 The Informing State

One group limited the role of the state to providing information and possibly education. Stakeholders who assigned responsibility mainly to individuals still emphasised that the state must provide the necessary information about risk factors and measures to prevent dementia. Although not viewing it as a state responsibility to prevent risky health behaviour, a Canadian bioethicist still saw the government as responsible for providing health education and information about dementia risk factors: ‘I don’t know that the government has a duty to prevent the conduct, but it has a duty to make sure that the conduct is informed and voluntary’ (CanEth2). This allocation of responsibility is advocated with slight variations by many stakeholders. A German patient representative, for example, argued: ‘I believe that raising awareness is a task for the state, but living it is an individual task in terms of autonomy’ (GerAdv1). As long as the state ensures citizens can make informed lifestyle choices and are aware of health risks and preventive measures, they are, as a German scientist elaborated, responsible for following a healthy, proactive lifestyle: ‘You can get the information you really need, so everybody has a responsibility on their own to change their habits to try to have a healthy lifestyle, but it’s also our institutional responsibility to educate people on how to manage a healthy lifestyle’ (GerSci4).

In this limited account of governmental responsibility, which still aligns with neoliberal conceptions of health policy (Ayo 2012), prevention remains a matter of individual choice or a citizen’s duty; the state is viewed as responsible only for educating the population and raising awareness about dementia prevention. Individuals are considered morally accountable for their lifestyle choices if they know that these behaviours involve health risks.

#### 4.4.2 The Enabling State

The other group of stakeholders defended a much more vital role for the state. Arguing that people need sufficient resources to adopt a healthy lifestyle and reduce the risk of developing dementia, they viewed it as the government’s responsibility to improve living conditions and reduce health disparities. A Canadian scientist acknowledged the relevance of personal responsibility in engaging in healthy activities but stressed the importance of resources and structural preconditions:

People have to take some kind of ownership over it at the individual level, but at the same time, we need to have the resources in place that people can take advantage of that. If people don't have access to healthy food, they can't do anything about that. That's got to come more from the top down. If they don't have places to go to be physically active, then that's more a municipal or provincial issue.

(CanSci5)

Acknowledging the social and environmental factors that affect individuals’ preferences and scope of action, these stakeholders proposed a shared understanding of responsibility, in which the state should not only inform but also empower people to make voluntary decisions. This interdependence of structural preconditions and personal engagement is illustrated, for instance, in an interview with a Canadian patient advocate who argued: ‘I think the state and society have to provide the means in which we can think about and engage with dementia prevention. And then the individuals have to feel like it’s accessible to them and then want to do it’ (CanAdv2).

To sum up, many stakeholders recognised the importance of social, economic and environmental preconditions for individual preventive efforts (Baum and Fisher 2014) and, therefore, emphasised the significance of population‐level political interventions to enable individuals to adopt healthy lifestyles for effectively advancing dementia prevention.

## 5 Discussion

Concepts of dementia prevention and associated responsibility ascriptions are shaped by new medical evidence regarding dementia risk factors as well as by normative assumptions about citizens’ duties, paradigms of successful ageing and health policy frameworks. Examining the underlying arguments for responsibility ascriptions in dementia prevention, our study shows that a simplistic contrast between positions emphasising personal responsibility for preventive lifestyle choices and those highlighting governmental responsibility for population‐level interventions falls short. Instead, both personal and governmental responsibilities for dementia prevention are justified by various epistemic, normative and pragmatic arguments.

The understanding of dementia prevention as a matter of individual lifestyle choices primarily rests on normative arguments that highlight the importance of personal autonomy and responsibility for preserving cognitive abilities. Some stakeholders view individuals’ lifestyle choices and health decisions as matters of personal freedom, asserting that the state should not interfere with choices that influence dementia risk. Others present personal efforts to reduce dementia risk as a social duty, citing the idea of a citizen’s obligation to remain productive and avoid dependence in old age (Cardona 2008; Katz and Peters 2008; van Dyk and Lessenich 2009). Additionally, some interviewed stakeholders understand individual actions pragmatically as the most promising approach to enhancing dementia prevention within current healthcare systems.

However, in contrast to current public media and health policy prevention discourses, which are characterised by a strong and partly exclusive emphasis on personal responsibility for healthy lifestyle choices and frame prevention as a functional necessity for reducing burdens on the welfare state (Chelberg 2025; Lawless and Augoustinos 2017; Petersen and Schicktanz 2021), stakeholders employ epistemic, pragmatic and normative justifications to defend nuanced concepts of joint responsibility for dementia prevention. Many interviewees do not adopt the oversimplified ascriptions of responsibility prevalent in the media; instead, they criticise holding the individual responsible for proactively preventing cognitive decline as medically insufficient and ethically problematic.

Many stakeholders argue that population‐level approaches are the most effective way to reduce dementia rates. Some highlight the relevance of socioeconomic factors that shape lifestyles and influence dementia risks (Wang et al. 2024; Wilson and Anstey 2024). Referencing the increased dementia risks faced by immigrants, ethnic minorities and Indigenous populations (Clarke et al. 2024), they advocate for a more comprehensive consideration of these factors in research and public health policies. Pointing to debate in health economics on the future impacts of demographic changes and the increasing burden of dementia (GBD 2019 Dementia Forecasting Collaborators 2022), others view strengthening population‐level approaches as an economic necessity to mitigate future healthcare costs. Moreover, some frame the need to strengthen population‐level prevention not only as essential for reducing dementia rates but also as crucial for avoiding the risk of stigmatisation, alleviating health inequalities and achieving equity.

This call for a more comprehensive approach to prevention aligns with emerging voices in medical and public health debates on dementia, who have recently begun to emphasise the need to combine individual‐level prevention with political population‐level efforts aimed at changing social, environmental, economic or legislative environments to improve living conditions and reduce inequalities (Daly 2024a; Walsh et al. 2022; Wilson and Anstey 2024). Although some population‐level approaches might initially appear politically challenging (e.g., outright bans or coercive measures), most interventions in practice aim to shift the environment in ways that make healthier choices more accessible and equitable. Some measures, such as smoking bans, taxation of alcohol and unhealthy foods, and initiatives to improve air quality, which are already partially implemented in varying degrees in certain countries, are considered cost‐effective and efficient for reducing dementia risk (Daly 2024b; Mukadam et al. 2024).

Interestingly, and contrary to our expectations, the significant differences in dementia policy between Canada, Germany and Switzerland are rarely reflected in the perspectives of the interviewed stakeholders. Although the Canadian dementia strategy emphasises prevention as a key goal, it plays only a secondary role in Germany and Switzerland (Andreoletti and Blasimme 2024; Jessen 2022). Yet nearly all interviewed stakeholders across all three countries highly value preventive measures. Furthermore, some stakeholders in each country emphasise the importance of population‐based approaches and view the state as responsible, whereas others defend a focus on individual prevention efforts. When explicitly asked about country‐specific differences, only a few stakeholders mention national peculiarities; only in Canada is the importance of prevention approaches tailored to minorities discussed more extensively. Additionally, the stakeholders’ views on dementia prevention do not seem to depend on their profession or position; any potential profession‐specific differences could not be systematically generalised. Despite the large sample size, some professional groups in individual countries were under‐represented, with only a few interviewees, and theoretical saturation was not fully achieved. Therefore, we have refrained from making a comparative generalisation here as well. The absence of clear country‐ and profession‐specific differences may indicate that the understanding of dementia and its prevention is influenced less by local care cultures and specific institutional contexts and more by political convictions, global scientific paradigms, conceptions of succesful ageing and health policy rationalities.

Above all, this study highlights the growing recognition of the social and political dimensions of dementia risk reduction among stakeholders in healthcare, patient advocacy and dementia research. From a policy standpoint, recognising dementia rates as reflections of environmental, economic and social living conditions would require comprehensive structural political interventions that go beyond health education and the moral appeal of individual behavioural prevention. We therefore suggest understanding dementia as deeply interconnected with social, economic and environmental conditions and argue for preventive strategies that extend beyond the health sector and address structural inequalities. In contrast to the tendency of increasing medicalisation (Conrad 2007), this view suggests a demedicalised understanding of prevention. The preservation of cognitive health would no longer be treated exclusively as a medical issue but would become a matter of social, environmental and educational policies.

We argue that future debates on effectively reducing dementia rates should not only focus on understanding the causal links between risk factors and dementia and the effectiveness of certain preventive measures but also on political discussions of the principles and rationalities guiding health policy. Whether the growing body of scientific evidence indicating that effective risk reduction depends on population‐level interventions will ultimately outweigh the prevailing neoliberal orientation of health policy remains a political question. Given that current health policy discourse often favours strategies focused on individual behavioural health promotion rather than population‐level interventions (Baum and Fisher 2014), scholars in medicine, public health, the social sciences and bioethics share a responsibility to engage not only with evidence on the effectiveness of risk reduction strategies but also to examine and debate the social, economic and political conditions, barriers and implications surrounding their implementation.

## Author Contributions

**Niklas Petersen:** writing – original draft, writing – review and editing, conceptualization, investigation, methodology, formal analysis. **Mattia Andreoletti:** investigation, formal analysis. **Alessandro Blasimme:** writing – review and editing, funding acquisition, investigation. **Cynthia Lazzaroni:** investigation, formal analysis. **Annette Leibing:** writing – review and editing, funding acquisition, investigation. **Silke Schicktanz:** writing – review and editing, conceptualization, funding acquisition, investigation, methodology, supervision.

## Funding

This research project was funded by an ERA‐NET ELSA NEURON grant (026) and the following national grants: Canada: Fonds de recherche du Québec—Santé (Grant No. 295645) and the Canadian Institutes of Health Research (ERN 174187); Germany: Federal Ministry of Education and Research (BMBF 01GP2120); Switzerland: Swiss National Science Foundation (SNSF 10NE17_199434).

## Ethics Statement

This study has received ethical approval from the relevant bodies in each country: ETH Zurich (EK 2022‐N‐08), University of Montreal (CF00147616 and CF00147827) and University Medical Center Göttingen (UMG 34/11/21).

## Consent

The authors have nothing to report.

## Permission to Reproduce Material From Other Sources

The authors have nothing to report.

## Supporting information


Supporting Information S1

## Data Availability

The data that support the findings of this study are available from the corresponding author upon reasonable request.

## Endnotes

^1^
Recently, the disease‐modifying medication lecanemab has received approval in several countries, including the United States and the European Union, but not in Canada and Switzerland. Despite demonstrating statistically significant slowing of cognitive decline, the clinical meaningfulness and cost‐benefit ratio remain highly contentious (Iacobucci 2025), and the introduction of this medication does not alleviate the urgency for preventive measures.

^2^
Although the evidence for the link between the preventive turn in dementia discourses and increasing stigmatisation of dementia is still limited, research on disease stigma shows that whereas stigma has declined for many diseases, negative associations tend to be attributed to behavioural health conditions and preventable diseases (Best and Arseniev‐Koehler 2023).

^3^
Interview codes refer to country and profession; see Table 1 for abbreviations.
