# Supplementary material for: Disentangling Responsibility: Perspectives on Dementia Prevention From Stakeholders in Canada, Germany and Switzerland
Source: Sociol Health Illn. 2025 Dec 8;48(1):e70126. doi: 10.1111/1467-9566.70126 (PMC12686603; doi:10.1111/1467-9566.70126)
Supplement: Supplementary file 1 — Supporting Information S1 [file SHIL-48-0-s001.docx]

Petersen, Niklas, Mattia Andreoletti, Alessandro Blasimme, Cynthia Lazzaroni, Annette Leibing, and Silke Schicktanz. 2026. “Disentangling Responsibility: Perspectives on Dementia Prevention From Stakeholders in Canada, Germany and Switzerland,” Sociology of Health & Illness: e70126.

**BEAD Project
Interview Guide Dementia Prevention**

*Short socio-demographic questionnaire*:

Stakeholder: ___

Age ____

Gender ____

Country ___________________________________

Profession _________________________________

Interviewer ________________________________

**Introduction**

1. We reached out to you for this interview because you are [activity of interviewee]. Could you please tell us more about this? How does your work or research relate to dementia prevention? [Prompting: Could you give us, please, a concrete example? Another example?]
2. Thinking about the last decade or so, has your opinion on dementia prevention changed? [Prompting: Why? If opinion has changed: What has influenced your change of opinion?]

a. How was dementia prevention discussed previously, and in what way?

b. Some researchers noted that, compared to other conditions – for example, cancer – dementia prevention only recently emerged. Do you have an idea why? [do not insist if they don’t know]

**Types of risk reduction**

1. As you probably know, a number of risk factors have been discussed in recent years; which are the most important ones in your opinion? [why?]
2. What could be done to address these risk factors? [general opinion]
3. What is your understanding of primary, secondary, and tertiary preventive measures, and how do you assess their effectiveness? [explore for each]

[If they are not familiar with these concepts, explain them: Primary prevention, for example, includes nutrition and cardiovascular training; secondary prevention involves pharmaceutical treatments and screening tests to detect disease at its earliest stages; and tertiary prevention could involve memory or physical training in nursing homes to slow down cognitive decline.]

3. There are various approaches to risk reduction and prevention.

a. How do you evaluate the potential of pharmaceutical therapies? What are their advantages and disadvantages? [explore + and -]

b. What role do drugs like Aducanumab play? [do not insist if unsure; if knowledgeable, please explore]

c. Another suggestion is lifestyle changes for dementia risk reduction. What are your thoughts on this?

d. Are there, in your opinion, any other methods of addressing dementia prevention apart from pharmaceutical and lifestyle interventions?

4. When comparing different approaches to dementia prevention, which ones would you prioritise and why?

5. What does “good ageing” mean to you personally?

**Differences**

1. Do you think there are individuals or groups within your country who are more at risk of developing dementia? [why? Explore!]

2. Are you aware of differences between countries on an international level? [if yes, explore]

3. Are there specific vulnerable groups, minorities, or communities that should be addressed in a targeted way when promoting dementia prevention, and if so, why?

4. What actions could be taken to address sociodemographic differences?

5. Do you believe these differences we have discussed raise any ethical questions?

**Concrete measures**

1. Do you believe prevention campaigns, including active ageing initiatives, are effective tools for prevention? Why (or why not)?
2. How do you evaluate the level of awareness among the general population or other stakeholder groups regarding active and healthy ageing in your country?
3. In your opinion, which approach to dementia prevention is currently being prioritised in your country? [What else?]

a. If not mentioned yet: The Lancet report on dementia prevention highlights less education as a risk factor. How do you assess this factor, and how is this addressed in the prevention strategy in your country?

b. If not mentioned yet: How are social asymmetries and social inequalities already considered in current prevention approaches? How do you assess these factors?

4. Who do you think is mainly responsible for dementia prevention, and in what way? In other words, which responsibilities fall to individuals, and which are the responsibilities of the State?

(If there is some preference for individual responsibility in their answer, please ask the following question: How do you evaluate the ethical concern that emphasising individual responsibility might weaken social solidarity with those in need of healthcare or social support?

5. Are you aware of currently used technologies such as apps, digital biomarkers, and others?

a. for prevention?

b. for early diagnosis?

c. Are they being already in use in your country? Please explain.

d. In your opinion, what is the value of such technologies?

e. Do you perceive any ethical challenges in the use of preventive technologies? [Explore]

**The future**

A final question about the future: What advancements do you anticipate in dementia prevention? [prompting: a. Will dementia prevention become more effective? b. What kinds of developments might facilitate this in the near future?]

Last question: Did we overlook any aspects in this discussion? Is there anything you would like to add that we haven’t considered?

THANK YOU SO MUCH FOR YOUR TIME.
